# Subjective cognitive impairment is related to work status in people with multiple sclerosis

**DOI:** 10.1016/j.ibneur.2022.10.016

**Published:** 2022-11-02

**Authors:** J. van Wegen, E.E.A. van Egmond, R.H.B. Benedict, E.A.C. Beenakker, J.J.J. van Eijk, S.T.F.M. Frequin, K. de Gans, O.H.H. Gerlach, D.A.M. van Gorp, G.J.D. Hengstman, P.J. Jongen, J.J.L. van der Klink, M.F. Reneman, W.I.M. Verhagen, H.A.M. Middelkoop, L.H. Visser, H.E. Hulst, K. van der Hiele

**Affiliations:** a Amsterdam UMC, location Vrije Universiteit Medisch Centrum, Department of Neurology, MS Center Amsterdam, Amsterdam Neuroscience, Amsterdam, the Netherlands; b Leiden University, Institute of Psychology, Health, Medical and Neuropsychology Unit, Leiden, the Netherlands; c University of Humanistic Studies, Utrecht, the Netherlands; d National Multiple Sclerosis Foundation, Rotterdam, the Netherlands; e Elisabeth-TweeSteden Hospital, Department of Neurology, Tilburg, the Netherlands; f University at Buffalo, Department of Neurology, New York, United States; g Medical Centre Leeuwarden, Department of Neurology, Leeuwarden, the Netherlands; h Jeroen Bosch Hospital, Department of Neurology, ’s-Hertogenbosch, the Netherlands; i St. Antonius Hospital, Department of Neurology, Nieuwegein, the Netherlands; j Groene Hart Hospital, Department of Neurology, Gouda, the Netherlands; k Zuyderland Medical Centre, Department of Neurology, Sittard-Geleen, the Netherlands; l Upendo MS Clinic, Boxtel, the Netherlands; m MS4 Research Institute, Nijmegen, the Netherlands; n University of Groningen, University Medical Centre Groningen, Department of Community & Occupational Medicine, Groningen, the Netherlands; o Tilburg University, Department of Social and Behavioral Sciences, Tranzo Scientific Center for Care and Welfare, Tilburg, the Netherlands; p Optentia, North West University of South Africa, Vanderbijlspark, South Africa; q Canisius-Wilhelmina Hospital, Department of Neurology, Nijmegen, the Netherlands; r Leiden University Medical Centre, Department of Neurology, Leiden, the Netherlands

**Keywords:** Multiple sclerosis, Cognitive impairment, Subjective cognitive difficulties, Work status, Work participation

## Abstract

**Background:**

Unemployment is common among people with multiple sclerosis (pwMS) and has been associated with subjective cognitive difficulties, specifically in memory, attention, and executive functioning. However, longitudinal research on subjective cognitive difficulties and employment is scarce.

**Objective:**

We investigated whether subjective cognitive impairment (SCI), based on the clinical cut-off score of the MS Neuropsychological Screening Questionnaire (MSNQ), was associated with work status and negative work events (NWE) at baseline and after 2 years. Moreover, we investigated whether four MSNQ subdomains were related to work status and NWE.

**Methods:**

287 participants (77.4% female, median age = 42 years) completed questionnaires on subjective cognitive functioning, depression, anxiety, and fatigue, and completed the Symbol Digit Modalities Test (SDMT). After baseline comparisons, logistic regression analyses were performed, with work status and NWE at baseline, and employment change and NWE change within 2 years after baseline as dependent variables. Independent variables included SCI and the MSNQ domains. Covariates anxiety, depression, fatigue, and SDMT were added.

**Results:**

SCI, depression and anxiety were associated with work status (*Nagelkerke R*^*2*^ = .286), but only SCI was associated with employment change (*Nagelkerke R*^*2*^ = .164). No predictors were associated with NWE at baseline or follow-up. In addition, no MSNQ subdomain was related to work status, employment change or NWE.

**Conclusion:**

Unemployed pwMS and pwMS with a deteriorated work status reported more cognitive difficulties after 2 years than employed pwMS or pwMS with a stable work status. In addition, depression, and anxiety were associated with work status.

## Highlights

• Subjective cognitive impairment is related to work status in MS.
• Depression and anxiety are associated with work status in MS.
• Subjective cognitive impairment predicts a deterioration in work status in two years.
• Subjective cognitive impairment was not related to negative work events.

## Background

Multiple sclerosis (MS) is one of the most common neurological diseases among young and middle-aged adults and often causes sensory deficits, reduced mobility and impaired cognition (Reich et al., 2018). Within 5 or 10 years after diagnosis, the majority of people with MS (pwMS) will develop work related issues as a consequence of MS or become unemployed (Uccelli et al., 2009). Reported unemployment rates among pwMS vary between 24% and 80% (Julian et al., 2008).

Impaired cognitive functioning is a common symptom of MS, affecting 43‐70% of pwMS (Chiaravalloti & DeLuca, 2008). Cognitive difficulties, particularly with executive functioning, information processing speed and memory, have been recurrently connected to unemployment and negative work events (NWEs) among pwMS (Benedict et al., 2014, Clemens and Langdon, 2018, Honan et al., 2015, Strober et al., 2014, Van Gorp et al., 2019). In fact, for pwMS whose physical abilities are still unaffected by MS, cognitive impairment alone can negatively affect work performance, which might eventually lead to unemployment (Baughman et al., 2015). Additionally, pwMS who experience NWEs are more likely to become unemployed (Frndak et al., 2015). In general, a distinction can be made between subjective cognitive difficulties and objective cognitive deficits, although this distinction is not always reflected in occupational literature and many studies only speak of cognitive symptoms (Vitturi et al., 2022). It is estimated that between 11.6% and 41.0% of pwMS experience subjective cognitive difficulties (Jelinek et al., 2019), which is elevated in comparison with healthy population (Benedict et al., 2004). They have the experience that their cognitive abilities have deteriorated, which often has a huge impact on their daily life, as normal daily activities now exceeding their cognitive abilities are hampered. Not all pwMS who experience cognitive difficulties also have measurable objective cognitive disturbances. Thus, for some pwMS, there is a discrepancy between how they experience their cognitive abilities and how they objectively perform on cognitive tests. In the literature, some studies report a relationship between subjective and objective cognitive difficulties (Benedict and Zivadinov, 2006, Nauta et al., 2019, Thomas et al., 2022), while others find no such correlation in pwMS (Benedict et al., 2003, Christodoulou et al., 2005). In these cases, subjective cognitive difficulties rather relate with other MS-related symptoms, such as depression, anxiety, or fatigue (Strober et al., 2016).

While more studies have investigated objective cognitive deficits and their influence on employment in MS, fewer studies have researched the influence of subjective cognitive difficulties on employment. Previous studies that have investigated the relationship between subjective cognitive difficulties and work status focused on self-reported general cognitive difficulties (D’hooghe et al., 2019, Julian et al., 2008, Kobelt et al., 2019, Kordovski et al., 2015, Roessler et al., 2001), or self-reported cognitive difficulties in one specific domain, such as self-reported difficulties with memory, executive functioning, attention, or concentration (Carrieri et al., 2014, Flensner et al., 2013, Honan et al., 2015, Moore et al., 2013, Van der Hiele et al., 2014, Van der Hiele et al., 2015a, Van der Hiele et al., 2015b). To our knowledge, no studies have considered subjective cognitive difficulties in several domains simultaneously. Therefore, the intention of the current study was to examine the association between subjective cognitive impairment and both work status and NWEs among pwMS. We considered cognitive difficulties in several domains independently to identify specific domains of subjective cognitive difficulties, if any, that relate to work status or NWEs. In addition, we examined whether these subjective cognitive difficulties could predict a deterioration in work status or an increase in NWEs within two years after baseline. Finally, because subjective cognitive difficulties are correlated with depressive symptoms, anxiety and fatigue (D’hooghe et al., 2019, Henneghan et al., 2017, Kinsinger et al., 2010, Lamis et al., 2018, Strober et al., 2016), the contribution of these covariates to work status and NWEs, in addition to the subjective cognitive difficulties, was also explored.

## Methods

### Design and procedure

For this study, empirical data from pwMS that were tested in light of the MS@Work study were used (Van der Hiele et al., 2015a, Van der Hiele et al., 2015b). The MS@Work study is a three-year longitudinal follow-up on factors related to work participation among people with relapsing-remitting MS. It included *N* = 287 pwMS and *N =* 134 healthy controls. The pwMS participating in the study were recruited from 16 MS outpatient clinics throughout the Netherlands. They were all diagnosed with relapsing-remitting MS, had no comorbid psychiatric or neurological disorders and were over 18 years old. They were either employed or within three years of their last employment. PwMS who were unable to speak Dutch were excluded from participation. For the healthy controls, the same inclusion criteria applied, except that the controls were not suffering from a chronic disorder.

Participants completed several online questionnaires every year for a period of three years. These questionnaires focused on demographic and disease characteristics, self-reported occupational and daily functioning, depression, anxiety, and the impact of fatigue. At their outpatient clinic, participants received both neurological and neuropsychological examinations. The data used for the current study are the baseline data and the two-year measure data.

### Participants

All pwMS participating in the MS@Work study (*N* = 287, 77.4% female) were included in this study. Of the pwMS who were employed at baseline (*N* = 250), 187 pwMS completed measurements after 2 years. They were divided into either having a stable employment status (SES) two years after baseline (*N* = 152) or having a deteriorated employment status (DES) two years after baseline (*N* = 35). Employed pwMS who completed questionnaires on NWEs after 2 years (*N* = 171) were divided into having a stable number of NWEs (stable NWE, *N =* 142) or having an increased number of NWEs (increased NWE, *N =* 15) after excluding self-employed pwMS (*N* = 14). Classification details can be found under “Employment”. The number of working hours per week ranged from 6 to 60. Two participants were enrolled in a part-time study.

### Measures

Table 1 shows an overview of the measures used for the data analysis. One additional variable was used for the baseline comparisons, namely the Expanded Disability Status Scale (EDSS), which was used to evaluate disability as a result of MS. Its score ranges from 0, meaning someone has a normal neurological examination, to 10, which means death due to MS (Kurtzke, 1983).

**Table 1:** Overview of the measures used for the analysis.

Variable	Measure
*Subjective cognitive functioning:*	
Multiple Sclerosis Neuropsychological Screeningn Questionnaire (MSNQ)	Score 0–4 for each question
* Domains*	*Scores per domain*
Attention and information processing	Score 0–12
Memory	Score 0–20
Other cognitive ability	Score 0–16
Personality and behaviour (social cognition)	Score 0–12
Subjective cognitive impairment (SCI)	0 (MSNQ < 27), 1 (MSNQ >= 27)
*Employment:*	
Work status	0 (no paid job), 1 (paid job, irrespective of number of working hours, parttime students and self-employment included)
Employment change	0 (stable work status after 2 years), 1 (deteriorated work status after 2 years)
Negative work events (NWE)	0 (no NWEs), 1 (>= 1 NWEs)
Negative work events change (NWE change)	0 (stable number of NWEs after 2 years), 1 (increased number of NWEs after 2 years)
*Covariates:*	
Hospital Anxiety and Depression Scale (HADS)	Depression total score (0–21) Anxiety total score (0–21)
Modified Fatigue Impact Scale (MFIS)	Total score (0–84)
Symbol Digit Modalities Test (SDMT)	Total number of correct digits within 60 s (0–110)

#### Subjective cognitive functioning

Subjective cognitive difficulties were assessed using the Multiple Sclerosis Neuropsychological Screening Questionnaire © (MSNQ). The MSNQ consists of 15 questions, developed to assess self-reported cognitive functioning and neuropsychiatric complaints in MS across several categories (Benedict et al., 2003), and has been used as such in several previous studies (Benedict et al., 2014, Campbell et al., 2017, D’hooghe et al., 2019, D’hooghe et al., 2020, Mäntynen et al., 2014, O’Brien et al., 2007). Although the MSNQ also includes questions about neuropsychiatric functioning (question 13, 14, 15, see Table 2), its focus lies on cognitive complaints and therefore we will refer to this measure as ‘subjective cognitive impairment’ (SCI) in this study. Participants were required to rate each question on a scale from 0 (never) to 4 (very often), resulting in a total score ranging from 0 to 60, in which a high score indicates more severe self-reported cognitive impairment. The items correlated strongly with one another in our sample: the lowest correlation was found between item 4 and item 14 (*r* = 0.124, *p* < .01) and the highest between item 8 and item 9 (*r* = 0.729, *p* < .01). Correlations of above .70 may indicate that two items assess the same construct, which is undesirable in a regression analysis (Meyers et al., 2013). Therefore, it was decided to combine the questions into separate variables by means of summation of their scores. Thus, new variables were created based on the four categories outlined by Benedict et al. (2003). See Table 2 for descriptions of the questions and their categories. Finally, pwMS were classified as having subjective cognitive impairment when their total MSNQ score was 27 or higher (Nauta et al., 2019).

**Table 2:** Descriptions of questions included in the MSNQ questionnaire (Benedict et al., 2003).

Number	Question	Category
1	Distractibility	Attention and information processing
2	Thoughts wandering off while listening to someone	Attention and information processing
3	Slow in solving problems	Attention and information processing
4	Forgetting appointments or commitments	Memory
5	Forgetting what one just read	Memory
6	Having trouble describing recently watched tv programs	Memory
7	Requiring that instructions get repeated	Memory
8	Needing to be reminded of tasks	Memory
9	Forgetting groceries or other tasks that were planned	Other cognitive ability
10	Struggle to answer questions coherently	Other cognitive ability
11	Struggle to follow two things at the same time	Other cognitive ability
12	Sometimes missing the point that someone is trying to make	Other cognitive ability
13	Sometimes struggle to control oneself	Personality and behaviour
14	Crying or laughing without clear reason	Personality and behaviour
15	Talking too much or being too focused on one’s own business	Personality and behaviour

#### Employment

For employment, four dichotomous variables were used. Firstly, work status was used to distinguish participants into being in paid employment and into being unemployed at baseline (self-employment included). Secondly, the variable ‘employment change’ was used to divide pwMS who were employed at baseline into having a stable employment status two years after baseline (SES) and having a deteriorated employment status 2 years after baseline (DES) (Morrow et al., 2010). A participant was regarded to be a part of the DES group if they had stopped working altogether as a result of MS or if their working hours decreased by at least 20% since baseline (Van Gorp et al., 2019). This second variable was introduced to detect more subtle changes in work status, on a longitudinal basis. The third variable is ‘negative work events’ (NWE), which is a measure of problems and/or accommodations in the work environment as a result of impaired functioning due to MS (Benedict et al., 2014). A participant scored 1 for NWE if they experienced one of the following NWEs in the past 3 months: decrease in scheduled work hours, verbal criticism for errors, formal discipline, mandatory additional retraining, asked to work extra hours to finish tasks, or diminution of job responsibilities (Van der Hiele et al., 2016). Since these experiences are not applicable for self-employed pwMS, these were excluded from the variable. The final employment variable is ‘negative work events change’ (NWE change). Participants scored 1 for NWE change when they reported a higher number of NWEs in the past 3 months at the 2-year measurement compared to baseline. Participants who had the same or fewer NWEs were classified as 0.

#### Covariates

Depression, anxiety and fatigue were included in the analysis as covariates, because they appear to be related to subjective cognitive difficulties (Strober et al., 2016). Depression and anxiety were assessed using the Hospital Anxiety and Depression Scale (HADS) (Zigmond & Snaith, 1983). This questionnaire, consisting of 14 questions, gives a total score for anxiety and a total score for depression. Higher scores indicate more symptoms of anxiety and depression. The impact of fatigue on daily functioning was assessed using the Modified Fatigue Impact Scale (MFIS) (Kos et al., 2003). This questionnaire consists of 21 questions with higher scores indicating a higher impact of fatigue on physical, cognitive, and psychosocial functioning. Objective cognitive functioning was added as an additional covariate, to evaluate whether this would alter the influence of subjective cognitive functioning. Objective cognitive functioning was measured by the Symbol Digit Modalities Test (SDMT). For this test, participants are asked to pair a sequence of random symbols with the correct single digit, based on a given key that indicates which digit (ranging from 1 to 9) should match which symbol (Smith, 1982). More correct pairs identified within 90 s result in a higher score, indicating better cognitive performance. Although the SDMT is a measure of information processing speed and is thus a simplistic measure of cognition, the test has been shown to reliably measure general cognition in pwMS (Benedict et al., 2017, Strober et al., 2009, Strober et al., 2019).

### Analysis

First, baseline comparisons and correlations were calculated for descriptive purposes. Independent sample t-tests (for normally distributed variables), Mann-Whitney tests (for not normally distributed variables) and chi-square independence tests (for categorical variables) were performed to determine which independent variables differed statistically significantly between the groups (employed vs. unemployed, SES vs. DES, NWE vs. no NWE and stable NWE vs. increased NWE). Subsequently, only statistically significant items (*p* < = .05) were added to eight logistic regression models. Predictors were regarded as borderline significant if their p-value was *p* < = .06. The logistic regression analyses used work status, employment change, NWE, and NWE change as outcome measures. The aim of the first analysis was to evaluate whether being classified as cognitively impaired based on the MSNQ is associated with the work status of pwMS. The second analysis was done in order to determine which, if any, subjective cognitive difficulties in a specific cognitive domain were associated with being employed or unemployed. For pwMS who were employed at baseline, the goal of the third and fourth analysis was to determine whether their work status would remain stable or deteriorate within two years after the baseline measure based on whether they have subjective cognitive impairment (SCI) on one hand, and their score on the MSNQ categories on the other hand. The fifth and sixth analysis aimed at determining whether pwMS experience NWEs based on the MSNQ categories and SCI respectively. The final two analyses were conducted to see if the predictors were able to distinguish pwMS into having a stable or increased number of NWE after 2 years. In all regression analyses, independent variables were added in blocks to the models. The first block consisted of demographics, i.e., sex, age, and educational level. The second block consisted of the covariates depression, anxiety, fatigue and objective cognitive functioning, and the final block consisted of SCI or the MSNQ categories. For an overview of the analyses, see Table 3. IBM SPSS for Mac (version 26) was used for the statistical analyses. It was decided not to correct for multiple testing given the exploratory nature of the analyses.

**Table 3:** Overview of the analyses performed.

Analysis	Type	Dependent variable	Predictors (if p <= .05)
1	Logistic regression	Work status (employed/unemployed)	Block 1: demographics Block 2: covariates Block 3: SCI
2	Logistic regression	Work status (employed/unemployed)	Block 1: demographics Block 2: covariates Block 3: MSNQ categories
3	Logistic regression	Employment change (SES/DES)	Block 1: demographics Block 2: covariates Block 3: SCI
4	Logistic regression	Employment change (SES/DES)	Block 1: demographics Block 2: covariates Block 3: MSNQ categories
5	Logistic regression	Negative work events (NWE/no NWE)	Block 1: demographics Block 2: covariates Block 3: SCI
6	Logistic regression	Negative work events (NWE/no NWE)	Block 1: demographics Block 2: covariates Block 3: MSNQ categories
7	Logistic regression	Negative work events change (stable NWE/increased NWE)	Block 1: demographics Block 2: covariates Block 3: SCI
8	Logistic regression	Negative work events change (stable NWE/increased NWE)	Block 1: demographics Block 2: covariates Block 3: MSNQ categories

## Results

### Baseline comparisons

Baseline comparisons between unemployed and employed (employed divided into SES group and DES group) pwMS are visualised in Table 4. There were no statistically significant differences in gender, age, educational level, and disease duration between employed and unemployed pwMS, and between the SES group and the DES group. Unemployed pwMS had a higher EDSS score than employed pwMS (t(254) = −2.96, *p* < .01). PwMS in the DES group had a higher EDSS score than pwMS in the SES group (t(40.8) = 2.57, *p* < .05). Furthermore, pwMS with a deteriorated work status two years after baseline, already worked statistically significantly fewer hours at baseline than pwMS whose work status remained stable over two years (t(178) = −2.36, *p* < .05), which could be related to their higher EDSS score at baseline.

**Table 4:** Baseline comparisons in demographics, MS-related characteristics, work measures, MSNQ items and covariates between SES (*N* = 152) and DES (*N* = 35), and between employed (*N* = 250) and unemployed (*N* = 37) pwMS.

	**MS, employed at baseline**			**MS, unemployed at baseline**	
	**SES**	**DES**	**Test statistic**^a^		**Test statistic**^b^
	%, mean (SD) or median (IQR), min-max	%, mean (SD) or median (IQR), min-max		%, mean (SD) or median (IQR), min-max	
**Demographics**					
Gender (% female)^d^	75.7%	82.9%	.83	78.4%	.03
Age^c^	42.0 (8.9), 21-63	43.3 (9.6), 21-60	.73	42.6 (9.5), 21-59	.41
Educational level^d^	Low (15.8%), middle (36.8%), high (47.4%)	Low (14.3%), middle (34.3%), high (51.4%)	.19	Low (13.5%), middle (56.8%), high (29.7%)	3.80
**MS-related characteristics**					
EDSS (range 0-10)^c^	1.9 (1.1), 0-6	2.6 (1.4), 0-6	2.57*	2.8 (1.4), 0-6	-2.96**
Disease duration (years)^e^	5.0 (8.0),.0-26	5.0 (9.0), 0-24	1903.5	4.0 (13.0),.0-18	3265.0
**Work measures**					
Number of work hours per week^c^	27.7 (11.4), 6-60	22.5 (11.2), 0-40	-2.36*	-	-
Type of work^d^	Mostly physical (11.0%), mostly mental (55.9%), both physical and mental (33.1%)	Mostly physical (8.0%), mostly mental (68.0%), both physical and mental (24.0%)	1.25	-	-
**MSNQ categories**					
Attention and information processing^c^	5.0 (2.0)	5.4 (1.9)	1.80	7.4 (2.2)	-5.77**
Memory^c^	6.8 (3.9)	8.7 (3.8)	2.66**	11.2 (4.0)	-5.46**
Other cognitive ability^c^	5.2 (2.9)	6.7 (2.8)	2.53*	8.7 (3.2)	-5.78**
Personality and behaviour^e^	3.0 (3.0)	3.0 (3.3)	2047.5*	3.5 (3.0)	3003.0*
Subjective cognitive impairment (SCI) (% MSNQ >= 27)^d^	20.0%	48.6%	12.22**	76.5%	32.91**
**Covariates**					
Anxiety^e^	5.0 (4.0)	5.0 (5.0)	2301.0	7.0 (5.0)	3023.5*
Depression^e^	2.0 (3.0)	3.5 (5.0)	1922.5*	6.0 (7.0)	1993.5**
Fatigue^c^	32.3 (14.6)	41.9 (17.2)	3.00**	48.7 (14.2)	5.10**
SDMT^c^	54.6 (8.1)	51.1 (8.5)	-1.99*	51.2 (9.8)	-1.51

SES: stable employment status; DES: deteriorated employment status; EDSS: Expanded Disability Status Scale; MSNQ: Multiple Sclerosis Neuropsychological Screening Questionnaire; SD: standard deviation; IQR: interquartile range.

a Test statistic comparing SES and DES groups.

b Test statistic comparing employed and unemployed pwMS.

c Independent samples t-test was used for comparing groups. Mean (SD) are reported.

d Chi-square independence test was used for comparing groups.

e Non-parametric Mann-Whitney test was used for comparing groups. Median (IQR) are reported.

* *p <.05.*

** *p <.01.*

For the MSNQ categories, unemployed pwMS had a statistically significantly higher score than employed pwMS for all MSNQ categories. PwMS in the DES group had a statistically significantly higher score than the SES group on all categories except for attention and information processing. Regarding SCI, 76.5% of unemployed pwMS had subjective cognitive impairment. For employed pwMS, this was the case for 20 % of the SES group and 48.6% of the DES group (27% for all employed pwMS). PwMS in the DES group were statistically significantly more often classified as having subjective cognitive impairment than pwMS in the SES group (Pearson χ^2^(1) = 12.22, *p* < .01). As for the covariates, there was a difference between employed and unemployed pwMS with regard to anxiety (U = 3023.5, *p* < .05), whereas there was no difference in anxiety scores between pwMS in the SES group and pwMS in the DES group. Reversely, for the SDMT score, there was a statistically significant difference between SES and DES groups (t(181) = −1.99, *p* < .05), but not between employed and unemployed pwMS. However, for depression and fatigue there were statistically significant differences between employed and unemployed pwMS (U = 1993.5, *p* < .01 and t(263) = 5.10, *p* < .01 respectively), and SES and DES groups (U = 1922.5, *p* < .05 and t(183) = 3.00, *p* < .01 respectively). Baseline comparisons for NWE and NWE change can be found in the appendix (Table 11 and Table 12 respectively).

### Correlations

Correlations between the total MSNQ score and the covariates are visualized in Table 5. Statistically significant correlations for employed but not for unemployed pwMS were found between MSNQ total and anxiety, MSNQ total and depression, and anxiety and fatigue (*p* > .05). Furthermore, the total MSNQ score was statistically significantly correlated with anxiety, depression, and fatigue, but only in employed pwMS (*r* = 0.52, *p* < .01; *r* = 0.45, *p* < .01; *r* = 0.61, *p* < .10 respectively). Finally, in our sample objective cognitive functioning (i.e., SDMT total) was not correlated with any of the other covariates, nor with the total MSNQ score.

**Table 5:** Spearman correlations between the total MSNQ score and the covariates anxiety, depression, fatigue, and SDMT for employed and unemployed pwMS.

Variable	MSNQ total		Anxiety		Depression		Fatigue		SDMT total	
	E	U	E	U	E	U	E	U	E	U
MSNQ total	–	–	0.52**	0.16	0.45**	0.33	0.61**	0.41*	-0.09	-0.24
Anxiety			–	–	0.59**	0.59**	0.45**	0.25	0.04	0.22
Depression					–	–	0.55**	0.47**	-0.12	-0.04
Fatigue							–	–	-0.11	-0.24
SDMT total									–	–

MSNQ: Multiple Sclerosis Neuropsychological Screening Questionnaire; SDMT: Symbol Digit Modalities Test; E: employed; U: unemployed.

* *p < .05.*

** *p < .01.*

### Logistic regression analyses

In total, eight logistic regression analyses were performed. The first two regressions used work status as dependent variable. None of the demographic variables gender, age, and educational level, as well as the SDMT score, differed statistically significantly between employed and unemployed pwMS. Thus, the first block of these regressions consisted of the covariates anxiety, depression and fatigue. This model was statistically significant (χ^2^(3) = 33.00, *p* < .001) and explained 22% of the variance (*Nagelkerke R*^*2*^ =.219). In this step of the regression, depression (*B* = −0.196, *p* = .008) and fatigue (*B* = −0.054, *p* = .001) were statistically significant correlates of work status. Adding subjective cognitive impairment in the second block improved the model (Block χ^2^(1) = 11.06, *p* < .001). This final model (Model χ^2^(4) = 44.05, *p* < .001) explained statistically significantly more variance than the first model (*Nagelkerke R*^*2*^ = .286). Anxiety (*B* = 0.146, *p* = .050), depression (*B* = −0.173, *p* = .025) and SCI (*B* = −1.601, *p* = .001) statistically significantly contributed to work status (see Table 6).

**Table 6:** Logistic regression model using work status as dependent variable and anxiety, depression, fatigue and SCI as predictors.

Included	B	SE	Wald	Odds ratio	*p*
Constant	4.038	0.728	30.748	56.712	**0.000**
**Covariates**					
Anxiety	0.146	0.075	3.844	1.158	**0.050**
Depression	-0.173	0.077	5.019	0.841	**0.025**
Fatigue	-0.033	0.017	3.613	0.967	0.057
Subjective cognitive impairment	-1.601	0.500	10.233	0.202	**0.001**

Model: *N* = 265, Nagelkerke R^2^ = .286, Hosmer and Lemeshow χ^2^(8) = 6.886 (*p* = .549), 86.8% correctly classified.

The second regression also used work status as dependent variable. Again, the first block of the regression consisted of the covariates anxiety, depression and fatigue, of which depression (*B* = −0.196, *p* = .008) and fatigue (*B* = −0.054, *p* = .001) were statistically significant predictors of work status. Adding the MSNQ categories in the second block, improved the model (Block χ^2^(4) = 14.22, *p* = .007). This final model (Model χ^2^(7) = 47.22, *p* < .000) explained statistically significantly more variance than the first model (*Nagelkerke R*^*2*^ = .305). Only depression statistically significantly contributed to work status (*B* = −0.181, *p* = .018). The contribution of attention and information processing was borderline significant (*B* = −0.313, *p* = .053) (see Table 7).

**Table 7:** Logistic regression model using work status as dependent variable and anxiety, depression, fatigue and the MSNQ categories as predictors.

Included	B	SE	Wald	Odds ratio	*p*
Constant	5.903	0.913	41.847	366.202	**0.000**
**Covariates**					
Anxiety	0.139	0.084	2.768	1.150	0.096
Depression	-0.181	0.077	5.569	0.835	**0.018**
Fatigue	-0.024	0.019	1.611	0.976	0.204
**MSNQ categories**					
Attention and information processing	-0.313	0.162	3.735	0.731	0.053
Memory	-0.008	0.092	0.007	0.992	0.932
Other cognitive ability	-0.139	0.135	1.065	0.870	0.302
Personality and behaviour	0.069	0.114	0.368	1.072	0.544

Model: *N* = 265, Nagelkerke R^2^ = .305, Hosmer and Lemeshow χ^2^(8) = 9.522 (*p* = .300), 89.1% correctly classified.

The third logistic regression analysis used employment change as dependent variable and thus only included pwMS who were employed at baseline. None of the demographic variables gender, age, and educational level differed statistically significantly between the SES and DES groups. Depression, fatigue, and the total SDMT score were added in the first block, since the groups did not differ statistically significantly in anxiety. This resulted in a statistically significant model (χ^2^(3) = 15.14, *p* = .002) explaining 13% of the variance (*Nagelkerke R*^*2*^ = .130). In this model, fatigue was a statistically significant predictor of employment change (*B* = 0.032, *p* = .032). Subjective cognitive impairment was added in the second block, which improved the model (Block χ^2^(1) = 4.26, *p* = .039). In this final model (see Table 8), SCI statistically significantly predicted a deterioration in work status after 2 years (*B* = 1.021, *p* = .039).

**Table 8:** Logistic regression model using employment change as dependent variable and depression, fatigue and SDMT as predictors.

Included	B	SE	Wald	Odds ratio	*p*
Constant	-0.314	1.466	0.046	0.730	0.830
**Covariates**					
Depression	0.074	0.075	0.994	1.077	0.319
Fatigue	0.015	0.017	0.816	1.016	0.366
SDMT	-0.044	0.025	2.944	0.957	0.086
Subjective cognitive impairment	1.021	0.494	4.273	2.777	**0.039**

Model: *N* = 181, Nagelkerke R^2^ = .164, Hosmer and Lemeshow χ^2^ (8) = 6.967 (*p* = .540), 80.7% correctly classified.

The fourth regression also used employment change as dependent variable. In the first block, covariates depression, fatigue and SDMT score were added to the model. This model was statistically significant (χ^2^(3) = 15.14, *p* = .002) and explained 13% of the variance (*Nagelkerke R*^*2*^ = .130). As for the MSNQ categories, memory, other cognitive ability, and personality and behaviour differed statistically significantly between the SES group and DES group (see Table 4). Thus, these variables were added in the second block. However, this step did not improve the model (Block χ^2^(3) = 1.49, *p* = .684). Thus, the first model was optimal. In this model (see Table 9), fatigue contributes statistically significantly to a deteriorated work status after 2 years (*B* = 0.032, *p* = .032).

**Table 9:** Logistic regression model using employment change as dependent variable and depression, fatigue and SDMT as predictors.

Included	B	SE	Wald	Odds ratio	*p*
Constant	-0.699	1.440	0.236	0.497	0.627
**Covariates**					
Depression	0.085	0.073	1.244	1.088	0.246
Fatigue	0.032	0.015	4.584	1.033	**0.032**
SDMT	-0.043	0.025	2.855	0.958	0.091

Model: *N* = 181, Nagelkerke R^2^ = .130, Hosmer and Lemeshow χ^2^ (8) = 9.159 (*p* = .329), 81.2% correctly classified.

The fifth and sixth logistic regression analyses used NWE as dependent variable. None of the demographic variables gender, age, and educational level differed statistically significantly between pwMS who did and did not experience NWEs at baseline. The two groups did not differ statistically significantly in SDMT scores, so anxiety, depression, and fatigue were added in the first block. This model was statistically significant (χ^2^(3) = 8.544, *p* = .036), explaining 6.0% of the variance in NWE (*Nagelkerke R*^*2*^ =.060), but no predictors were statistically significant. Then, subjective cognitive impairment was added in the second block, but this did not improve the model (Block χ^2^(1) = 0.777, *p* = .378). In the sixth analysis, all four MSNQ categories were added in the second step, but this did not improve the model either. Thus, for both analyses, the final model only included anxiety, depression, and fatigue (see Table 10).

**Table 10:** Logistic regression model using NWE as dependent variable and anxiety, depression, and fatigue as predictors.

Included	B	SE	Wald	Odds ratio	*p*
Constant	-2.405	0.507	22.458	0.090	**< 0.001**
**Covariates**					
Anxiety	0.052	0.058	0.795	1.053	0.373
Depression	0.072	0.069	1.109	1.075	0.292
Fatigue	0.012	0.014	0.749	1.012	0.387

Model: *N* = 218, Nagelkerke R^2^ = .060, Hosmer and Lemeshow χ^2^ (8) = 7.273 (*p* = .508), 78.9 % correctly classified.

The seventh and eighth logistic regression analysis used NWE change as outcome variable. None of the variables of interest (demographic variables, MSNQ categories, SCI, and covariates) differed statistically significantly between the two groups. Therefore, no analysis was performed.

## Discussion

This study aimed to evaluate whether SCI was associated with work status and/or NWE among pwMS. An association between SCI and work status was indeed found in our sample. We moreover attempted to identify subjective cognitive difficulties in a specific domain that related to work status or NWE among pwMS, however, no specific domain of cognitive difficulties could be identified. Additionally, our goal was to examine whether SCI and subjective cognitive difficulties in different domains could predict a deterioration in work status or an increase in NWE within 2 years after baseline. In our sample, SCI was predictive of a deterioration in work status, but not for an increase in NWEs. Since subjective cognitive difficulties are reported to be related to covariates such as anxiety, depression, and fatigue, the contribution of these factors was also investigated. We were able to confirm the relationship between depression and unemployment.

In the current relatively large sample of pwMS, depression, anxiety and fatigue were statistically significantly correlated with subjective cognitive difficulties for all employed pwMS. For unemployed pwMS, subjective cognitive difficulties were statistically significantly related to fatigue, but not to depression and anxiety. This latter finding is unexpected, since previous literature has repeatedly found a relationship between subjective cognitive difficulties and depression, both in employed and unemployed pwMS (D’hooghe et al., 2019, Henneghan et al., 2017, Kinsinger et al., 2010, Lamis et al., 2018). We speculate that this unexpected finding may have to do with the small number of unemployed pwMS in our sample (*N* = 37). Furthermore, in our sample subjective cognitive difficulties were not correlated with objective cognitive functioning, confirming findings of earlier studies (Benedict et al., 2003, Christodoulou et al., 2005).

### Subjective cognitive difficulties and work status

The results of the analyses with work status are in line with previous research finding a relationship between depression and work status (Dorstyn et al., 2019, Honarmand et al., 2011), and between fatigue and work status (Kobelt et al., 2019). After adding SCI to the model, fatigue ceased to be a statistically significant predictor of work status. This finding can be explained by the substantial correlations between fatigue and the MSNQ in the current sample, which demonstrate the intricate relationship between the concerned covariates and subjective cognitive difficulties. The finding that SCI was a statistically significant predictor for work status after accounting for the covariates, evidently demonstrates that when pwMS experience substantial cognitive difficulties, this jeopardizes their chances of being employed.

Anxiety was also statistically significantly associated with work status; however, the direction of this relation was positive, meaning that a higher score on an anxiety measurement led to higher odds of being employed. This finding is unexpected since the baseline comparisons show that unemployed pwMS had higher anxiety scores than employed pwMS, yet it is not a unique observation (Hartoonian et al., 2015). In our sample it can ostensibly be explained by the high correlations between anxiety and depression scores for both employed and unemployed pwMS. In order to evaluate whether this result can be ascribed to a reciprocal effect of anxiety and depression, two subsequent analyses were performed, one with anxiety, fatigue and SCI as predictors, and another one with depression, fatigue and SCI as predictors (results not reported). These analyses showed that when separating anxiety and depression into two regression analyses, neither anxiety nor depression remains a statistically significant predictor of work status.

In the second regression analysis, the MSNQ categories did not contribute statistically significantly to work status, while depression did. Whether depression has decreased the effect of the MSNQ categories on work status and if so, to which extent, remains debatable and requires more attention in future studies. The contribution of attention and information processing to work status was found to be borderline significant (*p* = .053). This finding corresponds with previous research stating that attention difficulties are commonly reported as a subjective cognitive difficulty in pwMS (Henneghan et al., 2017). It also matches previous research reporting that unemployed pwMS experience more distractibility and problems with sustained attention (Van der Hiele et al., 2015a, Van der Hiele et al., 2015b). Arguably, being able to pay attention to any given information is indispensable for properly retaining information and thus memory function, executive functioning and other cognitive functions (Gazzaniga et al., 2014). Attention problems can therefore have a negative impact on the daily activities of pwMS, among which their work activities. Future research should clarify and better illuminate whether such negative consequences can be traced back to MS-specific cognitive defects or to other factors.

We found a relationship between depression and work status, which confirms previous research (Dorstyn et al., 2019, Honarmand et al., 2011). Depressive symptoms often appear in pwMS and have extensive negative consequences for the daily activities and quality of life of pwMS (Benedict et al., 2005). In the current sample 31% of the participants score above the clinical cut-off score for depression, which is similar to previously reported prevalence of depression in MS (Boeschoten et al., 2017). This indicates that experiencing mild depressive symptoms affects work participation. Moreover, being employed is an essential contributor to one’s quality of life (Blustein, 2008, Gheaus and Herzog, 2016). For pwMS, being employed can be demanding both physically and mentally, and thus be a stress factor that may contribute to a depressed mood (Smith & Arnett, 2005). Therefore, it would be useful to investigate and treat depressive symptoms as early as possible in the course of the disease.

### Subjective cognitive difficulties and employment change

Fatigue statistically significantly predicted a deterioration in employment status after 2 years. After adding SCI to the model, SCI was a statistically significant predictor, while fatigue was not. This means that, when correcting for objective cognitive functioning (SDMT), subjective measures of cognition seem to be more important in explaining the variance in employment change. This suggests that subjective measures of cognition are informative for predicting a change in employment status, highlighting the need for attention to subjective cognitive functioning in pwMS.

### Subjective cognitive difficulties and NWEs

We found no statistically significant predictors of experiencing NWEs, perhaps due to the relatively small number of pwMS that experienced one or more NWEs at baseline (*N* = 49) compared to the number of pwMS that experienced no NWEs at baseline (*N* = 187). Another explanation could be that the scope of the variable NWE is too limited for this study. Participants were asked to report whether they experienced any of the NWEs in the past three months. Perhaps only asking about the past three months does not reveal enough problems at work. Furthermore, the six NWEs that are comprised by this variable are quite rigorous. There may be other NWEs, such as negative subjective experiences that have a large impact on the participant but are not reflected by this variable.

Finally, we were unable to perform analyses with the variable NWE change, possibly because this variable had an even more skewed distribution of subjects over the two groups (*N* = 15 participants had an increased number of NWEs after 2 years, *N* = 142 did not). Future research should replicate this study with a bigger sample size, so that relevant effects can be detected.

### Strengths and limitations

This study has several strengths and limitations that need to be highlighted. Strengths of the study include its large sample size and its longitudinal character. Participants completed the measurements again 2 years after baseline, allowing us to track changes in disability, subjective cognitive performance, and work status. Discovering predictors of changes in pwMS’ work situation especially benefits the search for accurate intervention methods to prevent a deterioration in work status and thereby to improve pwMS’ quality of life. A third strength of the current study is its focus on subjective cognitive difficulties, since subjective cognition in MS remains understudied and a general focus still lies upon objective cognitive performance. In particular, we looked at several domains of subjective cognitive performance among pwMS, while most studies investigating subjective cognitive difficulties in MS looked at general subjective cognitive abilities (D’hooghe et al., 2019, Julian et al., 2008, Kobelt et al., 2019, Kordovski et al., 2015, Roessler et al., 2001).

This study contains several limitations that need to be mentioned. First of all, we only used four measures of employment. Although the variable employment change was introduced to measure more subtle changes in work status over time, this is still too rigorous to capture the full scope of how subjective cognitive difficulties relate to the way participants function at work. NWE at baseline and at 2-year follow-up were added to generate more detailed information about this relationship, but these variables were unable to provide this information in our sample. Future attempts to explore the role of subjective cognitive difficulties in problems at work among pwMS should use samples with more equal distributions of participants among the groups, as well as additional measures, such as job type, the work ability index or the Work Role Functioning Questionnaire (WRFQ), that capture more aspects of functioning at work (Abma et al., 2013, Tuomi et al., 1991).

In addition to defining SCI based on the MSNQ total score, it was decided to combine the items into categories as outlined by Benedict et al. (2003). It should be noted, however, that unlike the MSNQ total score, these MSNQ categories have not been psychometrically evaluated. Additionally, the category “Other cognitive ability” compasses several cognitive domains that are undefined thus far. Hence, it can be argued that using the MSNQ for grouping subjective cognitive difficulties into cognitive domains requires additional research, and as such, the nature of this study with regards to the MSNQ is exploratory.

Finally, pwMS that participated in the MS@Work study are people with relatively mild MS and people with progressive forms of MS were not included. This means that they are fairly unaffected by the disease, which is also reflected by the relatively small percentage of pwMS that have a deteriorated employment status after 2 years (*N* = 35). This can possibly result in a distortion of the results and should be taken into consideration in future research on this topic.

## Conclusion

The current study found that, in line with previous literature, experiencing subjective cognitive difficulties is associated with unemployment and a deterioration in employment status after 2 years among relapsing-remitting pwMS. Furthermore, results of this study suggest that subjective difficulties with attention and information processing in MS are a candidate to focus on in future research due to its borderline significance level (0.053). All in all, the findings of this study emphasize the need for further research into subjective cognitive difficulties and their effect on work status among pwMS, as well as the interplay between depression, cognitive difficulties, and work participation.

## Compliance with ethical standards

This study was approved by an accredited METC (NL43098.008.12 1307, approved 12–02–2014) and was executed in accordance with the principles of the Declaration of Helsinki (World Medical Association, 2013). All subjects signed an informed consent form in advance of participation.

## Conflict of interest

The authors declare no conflict of interest.

## Figures and Tables

**Table 11:** Baseline comparisons in demographics, MS-related characteristics, work measures, MSNQ items and covariates between pwMS with one or more NWEs (*N* = 49) and with no NWEs (*N* = 201) at baseline.

	**MS, >=1 NWE at baseline**	**MS, no NWE at baseline**	**Test statistic**^a^
	%, mean (SD) or median (IQR), min-max	%, mean (SD) or median (IQR), min-max	
**Demographics**			
Gender (% female)^b^	77.6%	78.1%	.006
Age^c^	41.6 (11.2), 21-60	42.1 (9.0), 24-63	.29
Educational level^b^	Low (20.4%), middle (38.8%), high (40.8%)	Low (16.0%), middle (39.6%), high (44.4%)	.56
**MS-related characteristics**			
EDSS^c^	2.3 (1.1), 0-5	2.1 (1.3), 0-6	-1.00
Disease duration (years)^d^	4.5 (10.0), 0-24	5.0 (8.0), 0-31	3458.5
**Work measures**			
Number of work hours per week^c^	25.4 (10.9), 0-45	27.2 (11.5), 6-60	.97
Type of work^b^	Mostly physical (7.7%), mostly mental (64.1%), both physical and mental (28.2%)	Mostly physical (10.3%), mostly mental (55.1%), both physical and mental (34.6%)	1.04
**MSNQ categories**			
Attention and information processing^c^	6.0 (1.8)	5.1 (2.0)	-2.92**
Memory^c^	8.7 (4.0)	6.9 (3.8)	-2.74**
Other cognitive ability^c^	6.9 (2.9)	5.2 (2.8)	-3.61**
Personality and behaviour^d^	3.5 (3.0)	3.0 (2.0)	2997.5*
Subjective cognitive impairment (SCI) (% MSNQ >= 27)^b^	40.0%	23.1%	5.21*
**Covariates**			
Anxiety^d^	6.5 (6.0)	5.0 (4.0)	2961.0*
Depression^d^	4.5 (4.0)	2.0 (3.0)	2869.0**
Fatigue^c^	39.6 (14.4)	34.1 (14.7)	-2.26*
SDMT^c^	52.5 (10.0)	54.0 (8.8)	1.04

N: number of pwMS with available data for NWE; EDSS: Expanded Disability Status Scale; MSNQ: Multiple Sclerosis Neuropsychological Screening Questionnaire; SD: standard deviation; IQR: interquartile range.

*p <.05.

^**^p <.01.

a Test statistic comparing pwMS with one or more NWEs and pwMS with no NWEs at baseline.

b Chi-square independence test was used for comparing groups.

c Independent samples t-test was used for comparing groups. Mean (SD) are reported.

d Non-parametric Mann-Whitney test was used for comparing groups. Median (IQR) are reported.

**Table 12:** Baseline comparisons in demographics, MS-related characteristics, work measures, MSNQ items and covariates between groups with stable (*N =* 142) and increased number of NWEs (*N =* 15) 2 years after baseline.

	Stable NWE	Increased NWE	Test statistic^a^
	%, mean (SD) or median (IQR), min-max	%, mean (SD) or median (IQR), min-max	
**Demographics**			
Gender (% female)^c^	78.9 %	66.7 %	1.17
Age^b^	41.3 (9.0), 21–63	42.7 (8.6), 30–58	0.50
Educational level^c^	Low (14.1 %), middle (35.2 %), high (50.7 %)	Low (13.3 %), middle (26.7 %), high (60 %)	0.52
**MS-related characteristics**			
EDSS^b^	1.9 (1.1), 0–6	2.3 (1.6), 0–6	1.06
Disease duration (years)^d^	5.0 (7.0), 0–26	5.0 (12.0), 0–24	750.5
**Work measures**			
Number of work hours per week^b^	26.8 (11.6), 0–60	29.7 (8.4), 16–40	0.93
Type of work^c^	Mostly physical (11.5 %), mostly mental (55.7 %), both physical and mental (32.8 %)	Mostly physical (8.3 %), mostly mental (75 %), both physical and mental (16.7 %)	1.71
**MSNQ categories**			
Attention and information processing^b^	5.1 (2.0)	4.8 (1.8)	-0.50
Memory^b^	6.9 (3.8)	6.7 (3.5)	-0.26
Other cognitive ability^b^	5.4 (2.9)	4.3 (2.9)	-1.33
Personality and behaviour^d^	3.0 (2.0)	2.0 (4.3)	994.0
Subjective cognitive impairment (SCI) (% MSNQ >= 27)^c^	22.1 %	13.3 %	0.627
**Covariates**			
Anxiety^d^	5.0 (4.0)	6.0 (5.0)	875.0
Depression^d^	2.0 (3.0)	2.5 (5.0)	890.0
Fatigue^b^	32.5 (14.3)	33.1 (12.6)	0.17
SDMT^b^	55.1 (8.2)	53.5 (8.5)	-0.70

N: number of pwMS with available data for NWE change; EDSS: Expanded Disability Status Scale; MSNQ: Multiple Sclerosis Neuropsychological Screening Questionnaire; SD: standard deviation; IQR: interquartile range.

^a^Test statistic comparing pwMS with a stable and an increased number of NWEs compared to baseline.

^b^Independent samples t-test was used for comparing groups. Mean (SD) are reported.

^c^Chi-square independence test was used for comparing groups.

^d^Non-parametric Mann-Whitney test was used for comparing groups. Median (IQR) are reported.

*p < .05.

* *p < .01.

## Appendix

See Appendix Table 11, Table 12.

## References

[bib1] Abma F.I., Van der Klink J.J.L., Bültmann U. (2013). The work role functioning questionnaire 2.0 (Dutch Version): examination of its reliability, validity and responsiveness in the general working population. J. Occup. Rehabil..

[bib2] Baughman B.C., Basso M.R., Sinclair R.R., Combs D.R., Roper B.L. (2015). Staying on the job: the relationship between work performance and cognition in individuals diagnosed with multiple sclerosis. J. Clin. Exp. Neuropsychol..

[bib3] Benedict R.H.B., Rodgers J.D., Emmert N., Kiniger R., Weinstock-Guttman B. (2014). Negative work events and accommodations in employed multiple sclerosis patients. Mult. Scler. J..

[bib4] Benedict R.H.B., Zivadinov R. (2006). Predicting neuropsychological abnormalities in multiple sclerosis. J. Neurol. Sci..

[bib5] Benedict R.H.B., Munschauer F., Linn R., Miller C., Murphy E., Foley F., Jacobs L. (2003). Screening for multiple sclerosis cognitive impairment using a self-administered 15-item questionnaire. Mult. Scler..

[bib6] Benedict R.H.B., Wahlig E., Bakshi R., Fishman I., Munschauer F., Zivadinov R., Weinstock-Guttman B. (2005). Predicting quality of life in multiple sclerosis: accounting for physical disability, fatigue, cognition, mood disorder, personality, and behavior change. J. Neurol. Sci..

[bib7] Benedict R.H.B., Deluca J., Phillips G., LaRocca N., Hudson L.D., Rudick R. (2017). Validity of the symbol digit modalities test as a cognition performance outcome measure for multiple sclerosis. Mult. Scler. J..

[bib8] Benedict R.H.B., Cox D., Thompson L.L., Foley F., Weinstock-Guttman B., Munschauer F. (2004). Reliable screening for neuropsychological impairment in multiple sclerosis. Mult. Scler..

[bib9] Blustein D.L. (2008). The role of work in psychological health and well-being: a conceptual, historical, and public policy perspective. Am. Psychol..

[bib10] Boeschoten R.E., Braamse A.M.J., Beekman A.T.F., Cuijpers P., van Oppen P., Dekker J., Uitdehaag B.M.J. (2017). Prevalence of depression and anxiety in multiple sclerosis: a systematic review and meta-analysis. J. Neurol. Sci..

[bib11] Campbell J., Rashid W., Cercignani M., Langdon D. (2017). Cognitive impairment among patients with multiple sclerosis: Associations with employment and quality of life. Postgrad. Med. J..

[bib12] Carrieri L., Sgaramella T.M., Bortolon F., Stenta G., Fornaro L., Cracco A., Soresi S. (2014). Determinants of on-the-job-barriers in employed persons with multiple sclerosis: the role of disability severity and cognitive indices. Work.

[bib13] Chiaravalloti N.D., DeLuca J. (2008). Cognitive impairment in multiple sclerosis. The Lancet Neurology.

[bib14] Christodoulou C., Melville P., Scherl W.F., Morgan T., Macallister W.S., Canfora D.M., Krupp L.B. (2005). Perceived cognitive dysfunction and observed neuropsychological performance: longitudinal relation in persons with multiple sclerosis. J. Int. Neuropsychol. Soc..

[bib15] Clemens L., Langdon D. (2018). How does cognition relate to employment in multiple sclerosis? A systematic review. Mult. Scler. Relat. Disord..

[bib16] D’hooghe M.B., De Cock A., Benedict R.H.B., Gielen J., Van Remoortel A., Eelen P., Nagels G. (2019). Perceived neuropsychological impairment inversely related to self-reported health and employment in multiple sclerosis. Eur. J. Neurol..

[bib17] D’hooghe M.B., De Cock A., Van Remoortel A., Benedict R.H.B., Eelen P., Peeters E., D’haeseleer M., De Keyser J., Nagels G. (2020). Correlations of health status indicators with perceived neuropsychological impairment and cognitive processing speed in multiple sclerosis. Mult. Scler. Relat. Disord..

[bib18] Dorstyn D.S., Roberts R.M., Murphy G., Haub R. (2019). Employment and multiple sclerosis: a meta-analytic review of psychological correlates. J. Health Psychol..

[bib19] Flensner G., Landtblom A.-M., Söderhamn O., Ek A.-C. (2013). Work capacity and health-related quality of life among individuals with multiple sclerosis reduced by fatigue: a cross-sectional study. BMC Public Health.

[bib20] Frndak S.E., Irwin L.N., Kordovski V.M., Milleville K., Fisher C., Drake A.S., Benedict R.H.B. (2015). Negative work events reported online precede job loss in multiple sclerosis. J. Neurol. Sci..

[bib21] Gazzaniga M.S., Ivry R.B., Mangun G.R. (2014). Cognitive Neuroscience: The Biology of the Mind.

[bib22] Gheaus A., Herzog L. (2016). The goods of work (other than money!). J. Soc. Philos..

[bib23] Hartoonian N., Terrill A.L., Beier M.L., Turner A.P., Day M.A., Alschuler K.N. (2015). Predictors of anxiety in multiple sclerosis. Rehabil. Psychol..

[bib24] Henneghan A., Stuifbergen A., Becker H., Kullberg V., Gloris N. (2017). Perceived cognitive deficits in a sample of persons living with multiple sclerosis. J. Neurosci. Nurs..

[bib25] Honan C.A., Brown R.F., Batchelor J. (2015). Perceived cognitive difficulties and cognitive test performance as predictors of employment outcomes in people with multiple sclerosis. J. Int. Neuropsychol. Soc..

[bib26] Honarmand K., Akbar N., Kou N., Feinstein A. (2011). Predicting employment status in multiple sclerosis patients: the utility of the MS functional composite. J. Neurol..

[bib27] Jelinek P.L., Simpson S., Brown C.R., Jelinek G.A., Marck C.H., De Livera A.M., Weiland T.J. (2019). Self-reported cognitive function in a large international cohort of people with multiple sclerosis: associations with lifestyle and other factors. Eur. J. Neurol..

[bib28] Julian L.J., Vella L., Vollmer T., Hadjimichael O., Mohr D.C. (2008). Employment in multiple sclerosis: exiting and re-entering the work force. J. Neurol..

[bib29] Kinsinger S.W., Lattie E., Mohr D.C. (2010). Relationship between depression, fatigue, subjective cognitive impairment, and objective neuropsychological functioning in patients with multiple sclerosis. Neuropsychology.

[bib30] Kobelt G., Langdon D., Jönsson L. (2019). The effect of self-assessed fatigue and subjective cognitive impairment on work capacity: the case of multiple sclerosis. Mult. Scler. J..

[bib31] Kordovski V.M., Frndak S.E., Fisher C.S., Rodgers J., Weinstock-Guttman B., Benedict R.H.B. (2015). Identifying employed multiple sclerosis patients at-risk for job loss: when do negative work events pose a threat?. Mult. Scler. Relat. Disord..

[bib32] Kos D., Kerckhofs E., Nagels G., D’Hooghe B., Duquet W., Duportail M., Ketelaer P. (2003). Assessing fatigue in multiple sclerosis: Dutch modified fatigue impact scale. Acta Neurol. Belg..

[bib33] Kurtzke J.F. (1983). Rating neurologic impairment in multiple sclerosis: an expanded disability status scale (EDSS). Neurology.

[bib34] Lamis D.A., Hirsch J.K., Pugh K.C., Topciu R., Nsamenang S.A., Goodman A., Duberstein P.R. (2018). Perceived cognitive deficits and depressive symptoms in patients with multiple sclerosis: Perceived stress and sleep quality as mediators. Mult. Scler. Relat. Disord..

[bib35] Mäntynen A., Rosti-Otajärvi E., Koivisto K., Lilja A., Huhtala H., Hämäläinen P. (2014). Neuropsychological rehabilitation does not improve cognitive performance but reduces perceived cognitive deficits in patients with multiple sclerosis: a randomised, controlled, multi-centre trial. Mult. Scler. J..

[bib36] Meyers L.S., Gamst G., Guarino A.J. (2013). Applied Multivariate Research.

[bib37] Moore P., Harding K.E., Clarkson H., Pickersgill T.P., Wardle M., Robertson N.P. (2013). Demographic and clinical factors associated with changes in employment in multiple sclerosis. Mult. Scler. J..

[bib38] Morrow S.A., Drake A., Zivadinov R., Munschauer F., Weinstock-Guttman B., Benedict R.H.B. (2010). Predicting loss of employment over three years in multiple sclerosis: clinically meaningful cognitive decline. Clin. Neuropsychol..

[bib39] Nauta I.M., Balk L.J., Sonder J.M., Hulst H.E., Uitdehaag B.M.J., Fasotti L., de Jong B.A. (2019). The clinical value of the patient-reported multiple sclerosis neuropsychological screening questionnaire. Mult. Scler. J..

[bib40] O’Brien A., Gaudino-Goering E., Shawaryn M., Komaroff E., Moore N.B., DeLuca J. (2007). Relationship of the multiple sclerosis neuropsychological questionnaire (MSNQ) to functional, emotional, and neuropsychological outcomes. Arch. Clin. Neuropsychol..

[bib41] Reich D.S., Lucchinetti C.F., Calabresi P.A. (2018). Multiple sclerosis. New Engl. J. Med..

[bib42] Roessler R.T., Fitzgerald S.M., Rumrill P.D., Koch L.C. (2001). Determinants of employment status among people with multiple sclerosis. Rehabil. Couns. Bull..

[bib43] Smith, A. (1982). *Symbol digit modalities test: Manual*. Los Angeles, CA: Western Psychological Services.

[bib44] Smith M.M., Arnett P.A. (2005). Factors related to employment status changes in individuals with multiple sclerosis. Mult. Scler..

[bib45] Strober L.B., Binder A., Nikelshpur O.M., Chiaravalloti N., DeLuca J. (2016). The perceived deficits questionnaire: perception, deficit, or distress?. Int. J. MS Care.

[bib46] Strober L., Englert J., Munschauer F., Weinstock-Guttman B., Rao S., Benedict R.H.B. (2009). Sensitivity of conventional memory tests in multiple sclerosis: comparing the Rao brief repeatable neuropsychological battery and the minimal assessment of cognitive function in MS. Mult. Scler..

[bib47] Strober L., Chiaravalloti N., Moore N., DeLuca J. (2014). Unemployment in multiple sclerosis (MS): utility of the MS functional composite and cognitive testing. Mult. Scler. J..

[bib48] Strober L., DeLuca J., Benedict R.H.B., Jacobs A., Cohen J.A., Chiaravalloti N., LaRocca N.G. (2019). Symbol digit modalities test: a valid clinical trial endpoint for measuring cognition in multiple sclerosis. Mult. Scler. J..

[bib49] Thomas G.A., Riegler K.E., Bradson M.L., O’shea D.U., Arnett P.A. (2022). Relationship between subjective report and objective assessment of neurocognitive functioning in persons with multiple sclerosis. J. Int. Neuropsychol. Soc..

[bib50] Tuomi K., Ilmarinen J., Eskelinen L., Järvinen E., Toikkanen J., Klockars M. (1991). Prevalence and incidence rates of diseases and work ability in different work categories of municipal occupations. Scand. J. Work Environ. Health.

[bib51] Uccelli M.M., Specchia C., Battaglia M.A., Miller D.M. (2009). Factors that influence the employment status of people with multiple sclerosis: a multi-national study. J. Neurol..

[bib52] Van der Hiele K., Van Gorp D.A.M., Benedict R.H.B., Jongen P.J., Arnoldus E.P.J., Beenakker E.A.C., Visser L.H. (2016). Coping strategies in relation to negative work events and accommodations in employed multiple sclerosis patients. Mult. Scler. J. - Exp. Transl. Clin..

[bib53] Van der Hiele K., Middelkoop H.A.M., Ruimschotel R., Kamminga N.G.A., Visser L.H. (2014). A pilot study on factors involved with work participation in the early stages of multiple sclerosis. PLoS One.

[bib54] Van der Hiele K., Van Gorp D.A.M., Heerings M.A.P., van Lieshout I., Jongen P.J., Reneman M.F., Visser L.H. (2015). The MS@Work study: A 3-year prospective observational study on factors involved with work participation in patients with relapsing-remitting Multiple Sclerosis. BMC Neurol..

[bib55] Van der Hiele Karin, Van Gorp D., Ruimschotel R., Kamminga N., Visser L., Middelkoop H. (2015). Work participation and executive abilities in patients with relapsing-remitting multiple sclerosis. PLoS One.

[bib56] Van Gorp D.A.M., Van der Hiele K., Heerings M.A.P., Jongen P.J., Van der Klink J.J.L., Reneman M.F., Middelkoop H.A.M. (2019). Cognitive functioning as a predictor of employment status in relapsing-remitting multiple sclerosis: a 2-year longitudinal study. Neurol. Sci..

[bib57] Vitturi B.K., Rahmani A., Dini G., Montecucco A., Debarbieri N., Sbragia E., Durando P. (2022). Occupational outcomes of people with multiple sclerosis: a scoping review. BMJ Open.

[bib58] World Medical Association (2013). World Medical Association Declaration of Helsinki: Ethical Principles for Medical Research Involving Human Subjects. JAMA: J. Am. Med. Assoc..

[bib59] Zigmond A.S., Snaith R.P. (1983). The hospital anxiety and depression scale. Acta Psychiatr. Scand..

